# Extracorporeal cytokine adsorption as an alternative to pharmacological inhibition of IL-6 in COVID-19

**DOI:** 10.1186/s13054-020-03238-1

**Published:** 2020-08-20

**Authors:** Alexander Supady, Daniel Duerschmied, Christoph Bode, Marina Rieder, Achim Lother

**Affiliations:** 1 Department of Medicine III (Interdisciplinary Medical Intensive Care), Medical Center – University of Freiburg, Faculty of Medicine, University of Freiburg, Freiburg, Germany; 2 Department of Cardiology and Angiology I, Heart Center, University of Freiburg, Hugstetter Strasse 55, 79106 Freiburg, Germany; 3 Heidelberg Institute of Global Health, University of Heidelberg, Heidelberg, Germany; 4 Institute of Experimental and Clinical Pharmacology and Toxicology, Faculty of Medicine, University of Freiburg, Freiburg, Germany

With great interest we read the article by Convertino et al. discussing potential treatment targets for pharmacological immunomodulation in coronavirus disease 2019 (COVID-19) with acute respiratory distress syndrome (ARDS) [1]. We would like to add to the debate some thoughts about cytokine adsorption, which was mentioned only in passing in this discussion.

Following initial reports describing Interleukin-6 (IL-6) as a predictive factor for a negative outcome, extracorporeal cytokine adsorption was discussed as a possible treatment option for severe COVID-19 cases. Initial experience at our center using the CytoSorb® device (CytoSorbents Europe, Berlin, Germany) in combination with veno-venous extracorporeal membrane oxygenation (V-V ECMO) in severe COVID-19 yielded promising results; cytokine adsorption resulted in a more pronounced decrease of IL-6 after initiation of V-V ECMO as compared to patients treated without cytokine adsorption [2].

The use of the term “cytokine storm” in the context of COVID-19 has been challenged, though. While elevated levels of IL-6 are associated with poor outcome, absolute levels in these cases are rather moderately elevated in comparison to other forms of ARDS with extensive IL-6 increases [3]. Inflammatory dysregulation in severe cases is probably more complex and does not only go along with an upregulation of interleukins or TNF-α but also with an impaired interferon response [4].

A major advantage of extracorporeal cytokine adsorption over the other therapeutic approaches discussed in this debate is that it does not selectively block a specific receptor or signal transduction cascade, but it rather reduces particularly elevated concentrations of various inflammatory mediators such as interleukins, TNF-α, and also interferons; these factors have both pro- and anti-inflammatory functions. Only mildly elevated, physiological, or even decreased concentrations are not relevantly altered; thus, over-suppression of the immune response may be prevented [5]. Furthermore, cytokine adsorption can be better controlled than the other mentioned treatment options—it can be terminated at any time without any specific after-effect. These two aspects may be particularly relevant, e.g., in the case of bacterial superinfection in severe COVID-19 when an adequate immune response is required.

In conclusion, we recommend a cautious approach to intervention or “modulation” in the immune response in COVID-19 patients as long as the pathophysiological background remains to be unveiled. All interventions discussed in this debate should be considered experimental and therefore applied and evaluated within clinical trials.

## Data Availability

Not applicable.
